# The Modelled Population Obesity-Related Health Benefits of Reducing Consumption of Discretionary Foods in Australia

**DOI:** 10.3390/nu12030649

**Published:** 2020-02-28

**Authors:** Anita Lal, Anna Peeters, Vicki Brown, Phuong Nguyen, Huong Ngoc Quynh Tran, Tan Nguyen, Utsana Tonmukayakul, Gary Sacks, Hanny Calache, Jane Martin, Marj Moodie, Jaithri Ananthapavan

**Affiliations:** 1 Deakin Health Economics, Institute for Health Transformation, Deakin University, Geelong, VIC 3220, Australia; 2 Global Obesity Centre (GLOBE), Institute for Health Transformation, Deakin University, Geelong, VIC 3220, Australia; 3 Obesity Policy Coalition, Cancer Council Victoria, Melbourne, VIC 3004, Australia

**Keywords:** obesity, discretionary foods, sugar-sweetened beverages, healthcare costs

## Abstract

Over one third of Australians’ daily energy intake is from discretionary foods and drinks. While many health promotion efforts seek to limit discretionary food intake, the population health impact of reductions in the consumption of different types of discretionary foods (e.g., sugar-sweetened beverages (SSBs), confectionery, sweet biscuits) has not been quantified. This study estimated the potential reductions in body weight, obesity-related disease incidence, and healthcare cost savings associated with consumption of one less serving per week of different discretionary foods. Reductions in the different types of discretionary food were modelled individually to estimate the impact on energy consumption and population body weight by 5-year age and sex groups. It was assumed that one serving of discretionary food each week was replaced with either a serving of fruit or popcorn, and a serving (375 mL) of SSBs was replaced with coffee, tea, or milk. Proportional multi-state multiple-cohort Markov modelling estimated likely resultant health adjusted life years (HALYs) gained and healthcare costs saved over the lifetime of the 2010 Australian population. A reduction of one serving of SSBs (375 mL) had the greatest potential impact in terms of weight reduction, particularly in ages 19–24 years (mean 0.31 kg, 95% UI: 0.23 kg to 0.37 kg) and overall healthcare cost savings of AUD 793.4 million (95% UI: 589.1 M to 976.1 M). A decrease of one serving of sweet biscuits had the second largest potential impact on weight change overall, with healthcare cost savings of $640.7 M (95% CI: $402.6 M to $885.8 M) and the largest potential weight reduction amongst those aged 75 years and over (mean 0.21 kg, 95% UI: 0.14 kg to 0.27 kg). The results demonstrate that small reductions in discretionary food consumption are likely to have substantial health benefits at the population level. Moreover, the study highlights that policy responses to improve population diets may need to be tailored to target different types of foods for different population groups.

## 1. Introduction

An unhealthy diet is a major risk factor for obesity, and is a significant contributor to the burden of disease in Australia [1]. Over one third (35%) of the total daily energy intake of Australians is consumed from discretionary foods and drinks [2], that are not necessary to provide the nutrients that the human body needs. For Australians aged 14–18 years, the total daily energy intake from discretionary foods is even higher at 41% [2]. Higher rates of discretionary food consumption are also reported amongst lower socioeconomic position (SEP) groups, compared to higher SEP groups [3]. 

Discretionary foods tend to be energy dense, have low levels of essential nutrients, and are often eaten as snacks instead of food that is more nutritious. Examples include sweet biscuits, cakes, frozen milk products, pies, chocolate, commercial burgers, commercially fried foods, salty snack foods, sugar sweetened beverages (SSBs), and alcoholic drinks [4]. The Australian Dietary Guidelines (ADGs) recommend that Australians eat these foods less often and in smaller amounts to enable daily nutrient requirements to be met while staying within the recommended energy and sugar intake limits [4]. Discretionary food consumption is therefore an important target for public health policy that aims to reduce overweight and obesity and associated diseases. 

Previous studies investigating the health impacts of policies to reduce consumption of discretionary foods have shown that taxes, package size limits and restricting marketing of discretionary foods and drinks would result in considerable health benefits [5,6,7,8,9,10,11,12,13,14,15,16]. These studies have focused either on SSBs or on discretionary foods in general. In order to inform health promotion policy in different settings relevant to different populations, additional information on how the consumption of various discretionary foods differ by age and the estimated health impact of reductions in consumption of different types of discretionary food are required. Moreover, previous studies have focused on health outcomes reported as changes in the intermediate risk factor (e.g., weight, body mass index (BMI), blood pressure, or cholesterol) [17,18] and generic long term health outcomes such as health adjusted life years (HALYs). Whilst these outcomes are important, reporting the impact of reductions in discretionary foods on the number of cases of specific diseases prevented may increase saliency for policy makers and the general public.

This paper aimed to estimate the potential population health impact and healthcare cost savings of reductions of different types of discretionary foods in Australia.

## 2. Method

### 2.1. Overview of Methods

Consumption of each discretionary food group was estimated for consumers by age and sex. The replacement of a single serving each week of commonly consumed sweet snack foods replaced with fruit, salty snacks replaced with popcorn, and SSBs replaced with coffee and tea for adults and plain low fat milk for children was modelled by estimating the net reduction in kilojoules (kJ) and the resultant reduction in body weight. Changes in body weight were used to estimate the long-term reduction in obesity-related diseases and healthcare cost savings. 

### 2.2. Current Consumption

Current consumption of discretionary foods was obtained by categorising the items from the Australian Health Survey (AHS) 2011–2012 [19] under the broad categories in Table 1. The AUSNUT classification system was used to group foods from the survey [20] (Table S1). Eight types of discretionary foods and drinks from the ADGs were chosen based on the snack foods commonly consumed in Australia [4]. These fell under the broad categories of cakes, sweet biscuits, salty snacks, chocolate, frozen milk products, muesli bars, confectionery and SSBs. Each discretionary food item consumed was classified under our broad discretionary food types and the mean daily kJs consumed were then calculated based on kJ content per 100 g from the AUSNUT 2011–2013 data files [21]. 

Data from the AHS 2011–2012 were analysed to determine the mean daily consumption of each discretionary food type and the proportion of the population who had consumed these foods by sex and age groups (2 to 4, 5 to 12, 13 to 18, 19 to 24, 10 year age groups from 25 to 74, and 75 to 100 years) [19]. The mean daily kJ consumed for each discretionary food was calculated based on the mean daily intake over the two days surveyed. The proportion of tea and coffee drinkers was also extracted from the AHS (2011–2012) [19].

### 2.3. Scenarios Modelled 

In the primary analysis (scenario 1), consumers of discretionary foods substituted one serving of discretionary food per week with a healthier substitute based on the LiveLighter^®^ health promotion campaign [22]. Serving sizes were based on the ADGs [4] (Table 1). For example, one serving size of cake (40 g) was replaced with one serving size of fruit (150 g). For all sweet discretionary snack food categories, the healthier substitute was one serving of fruit per week, and for salty snacks, the healthier substitute was one serving of popcorn (15 g). For SSBs, the healthier substitute was coffee and tea (250 mL) for adults and low fat plain milk (250 mL) for those aged under 18 years The mean kJ in a serving size of discretionary foods, tea, and coffee were calculated based on kJ content per 100 g from the AUSNUT 2011–2013 data files [21]. The mean kJ in seven commonly consumed examples of fresh servings of fruit (red apple, banana, pear, orange, kiwi fruit, plum, and apricot) and milk (cow’s or soy) was obtained from the FSANZ NUTTAB online [23]. The mean kJ in a serving of popcorn was obtained from the Live Lighter^®^ website [22].

A second scenario (scenario 2) modelled the impact of consumers reducing one serving of discretionary food or drink per week without substitution to other foods/drinks.

### 2.4. Assessment of Health Benefits

#### 2.4.1. Overview

The ACE-Obesity Policy model, full methods published elsewhere [5,24], estimated differences in HALYs pre- and post- reduction of the discretionary items. These differences were based on predicted variations in nine diseases related to obesity. Changes in BMI were modelled based on projected reductions in energy intake (kJ/day).

#### 2.4.2. Effect of the Reduction in Consumption of Discretionary Foods on Body Weight

The mean kJ per serve was converted to an equivalent daily reduction. The kJ reduction was converted to a change in body weight based on previously published equations for adults [25] and children [26]. These changes in weight were converted to changes in BMI using average Australian height and weight by sex and single-year age groups up to 19 years and 5-year age groups thereafter obtained from the AHS 2011–2012 [19]. It was assumed that the reduction in energy intake was as a result of policies that were in steady state and weight loss had already occurred. The evidence indicates that the estimated weight loss will occur approximately three years after policy implementation [24].

#### 2.4.3. Modelling Obesity-Related Health Outcomes

The assessment of health impact of each scenario was undertaken using a previously developed model. The model is a multi-state, multiple cohort life table Markov model that estimates how a change in the distribution of the prevalence of overweight and obesity caused by a reduction in BMI impacts the epidemiology of nine obesity-related diseases, long term health status measured as HALYs and healthcare costs. HALYs are estimated using disease specific disability weights from the Global Burden of Disease 2010 study [27] and disutility attributable to elevated BMI in childhood [28].

The reductions in discretionary choices were modelled as a population-based intervention, assuming maintenance of effect over the cohort’s lifetime, estimating the health and cost effects among all ages (2–100 years) for the 2010 Australian population.

The diseases included in the model are diabetes mellitus, ischemic heart disease, stroke, hypertensive heart disease, colorectal cancer, breast cancer, endometrial cancer, kidney cancer, and osteoarthritis of the knee and hip. Each disease was modelled with four health states (healthy, diseased, dead due to disease, and dead due to other causes). Population impact fractions, calculated using relative risk of diseases related to BMI, were used to quantify the proportional reduction in disease incidence that would occur if a population were subject to the changes in BMI exposure compared to a counterfactual risk exposure (i.e. the BMI profile in the 2010 population). Changes in disease incidence result in changes in disease prevalence, and disease-specific mortality and morbidity.

#### 2.4.4. Assessment of Healthcare Cost Savings

A health sector perspective was adopted to calculate healthcare cost-savings from diseases averted as a result of reduced consumption of discretionary foods. Costs were based on the Disease Costs and Impact Study 2001 data from the Australian Institute of Health and Welfare (AIHW) [29], inflated to 2010 Australian Dollars using AIHW health price inflation values [30]. Costs included hospital services, out of hospital medical services, pharmaceuticals, and health professional services.

Future cost savings and benefits (HALYs) were discounted at 3% per year as per previous cost-effectiveness analyses for prevention interventions in Australia and are presented in 2010 values [5,14,31]. Incident cases of diseases saved were not discounted.

#### 2.4.5. Uncertainty and Sensitivity Analyses

Monte-Carlo simulation was employed to estimate the impact of uncertainty around input parameters (kJ per serve) on the main outcome measures (Table 1). Means and 95% uncertainty intervals for the modelled scenarios’ effects on weight, incident cases of disease, HALYs, and health care cost savings are reported based on 2000 iterations of the model using Ersatz version 1.3 software [32].

## 3. Results

Of the discretionary foods and drinks examined, SSBs were consumed by the highest percentage of the population (40%). The percentage of the population consuming SSBs was highest in those aged 13 to 18 years (64%), followed by 19 to 24 years olds (58%) and 4 to 12 years old (55%). Of the discretionary foods, sweet biscuits had the highest percentage of consumers at ages 2 to 4 years (44%) followed by ages 75 years and over (42%), 5 to 12 years (41%), and 65 to 74 years (37%). The highest percentage of consumers of salty snacks, confectionery, muesli bars, and frozen milk products were in people aged 18 years and under. The highest percentage of consumers of cake were 5 to 13 years olds (28%) (Table 2).

In all age groups, cake accounted for the highest mean daily kJ consumed, with estimates of between 1385 kJ to 2056 kJ. The highest consumers were in ages 25 to 34 years 2056 kJ (95% CI: 2005 kJ to 2109 kJ) followed by 13 to 18 years (2011 kJ (95% CI 1961 kJ to 2063 kJ). Frozen milk products had the second highest mean daily kJ intake at 1160 kJ in ages 19 to 24 years (95% CI: 1091 kJ to 1233 kJ), followed by chocolate in 13 to 18 years olds with a mean daily intake of 1086 kJ (95% CI: 1052 kJ to 1118 kJ) (Table 2).

### 3.1. Scenario 1

Under scenario 1, when one serve of sweet discretionary food was replaced with fruit, salty discretionary food was replaced with popcorn and SSBs were replaced with coffee, tea, or milk, a reduction of one serving of SSBs was estimated to result in the greatest decreases in mean weight overall in the population of 0.21 kg (95% CI: 0.16 kg to 0.25 kg). The biggest decrease in weight was in ages 19 to 24 years of 0.31 kg (95% CI: 0.23 kg to 0.37 kg) followed by ages 25 to 34 of 0.26 kg (95% CI: 0.20 kg to 0.31 kg). A reduction in sweet biscuits was estimated to result in the second highest decrease in weight overall of 0.12 kg (95% CI: 0.08 kg to 0.17 kg) with the highest decrease in weight in ages 75 years and over of 0.21 kg (95% CI: 0.14 kg to 0.27 kg), followed by ages 65 to 74 years of 0.18 kg (95% CI: 0.12 to 0.23) (Table 3).

Replacing one serve of SSBs with coffee, tea, or milk per week was estimated to result in both the highest healthcare costs savings of 793.4 M (95% UI: 589.1 M to 976.0 M) and HALY gains of 76,441 (95% UI: 57,214 to 94,597) over the lifetime. When one serve of discretionary food was replaced with a healthier option, sweet biscuits had the highest HALY gains of 66,550 (95% UI: 41,702 to 91,563) and healthcare cost savings of $640.7 M, (95% UI: 402.6 M to 885.8 M), followed by cakes with 44,711 HALYs gained (95% UI: 3907 to 90,169) and $447.1 M healthcare cost savings (95% UI: 38.3 M to 903.2 M) (Table 4).

The largest reductions in obesity related disease were predicted for cases of diabetes, osteoarthritis, and ischaemic heart disease (Table 4).

### 3.2. Scenario 2

Under scenario 2 with no substitutions, the health care cost savings and HALYs gained for the reductions in foods are approximately 50% greater. A reduction of one serve of sweet biscuits was estimated to result in the greatest decreases in weight overall, total HALY gains (134,168) (95% UI: 110,377 to 159,044) and reductions in incident cases of ischaemic heart disease (23,768) (95% UI: 19,715 to 27,808) and stroke (9829) (95% UI: 7100 to 12,815) and breast cancer (2102) (95% UI (944 to 3356). This was followed by SSBs, estimated to result in 129,997 HALYs gained, (95% UI: 92,532 to 173,013) and the prevention of the highest incident cases of type 2 diabetes (53,768) (95% UI: 36,767 to 73,561) and osteoarthritis (29,258) (95% UI: 18,384 to 42,890). The largest reductions in obesity-related disease were predicted for cases of diabetes, osteoarthritis, and ischaemic heart disease. The detailed results of scenario 2 can be found in the supplementary materials (Table S2). 

## 4. Discussion

In our study, we estimated that weekly substitution of one serve of SSBs with coffee, tea, or milk, or one serve of discretionary foods with a serve of fruit or popcorn could result in small reductions in population weight which translates to large improvements in the longer term health outcomes for the Australian population and substantial healthcare cost savings to the government. SSBs had the highest percentage of weekly consumers in all age groups, resulting in the highest overall weight reduction and health benefits from reduced consumption. Of the discretionary foods, sweet biscuits had the highest percentage of consumers in the youngest and oldest ages. Across all foods and SSBs, the highest reductions in obesity-related disease were predicted to be cases of type 2 diabetes, followed by osteoarthritis and ischemic heart disease.

Various factors impact on our results. Our assumptions regarding the foods/drinks that will be substituted for one serve of discretionary food and drink has the largest impact. The largest reductions in disease were predicted when a serve of SSB was substituted with coffee, tea, or milk. However, these reductions would have been even larger if the substitute was water with zero kJ (scenario 2). In addition to the kJ associated with substitution, the other factors that impacted the results include the proportion of the population at various age groups who are consumers of the food/drink and the relative risk of disease at various ages. For example, in scenario 2 (where there is no substitution), we found much higher incident cases prevented for stroke, ischaemic heart disease and colon, breast, kidney, and endometrial cancers by a reduction in sweet biscuits when compared to SSBs. However, the number of incident osteoarthritis cases prevented was higher for a reduction of SSBs than sweet biscuits, due to higher rates of consumption in younger ages and because there is a constant relative risk of osteoarthritis from age 30 years onwards [27].

This is the first study to report a comparative assessment of the potential impact of reductions in individual servings of discretionary foods and drinks on the overall health outcomes and the incidence of obesity-related diseases. Previous studies examining the impact of discretionary foods have not reported the relative impact of specific foods and serving sizes that are relatable and easily understood by the general population. Reducing one serve of SSBs or discretionary food per week is a tangible goal with potentially large health impacts. A review of discrete strategies to reduce intake of discretionary foods found that substituting discretionary choices for high fibre snacks, fruit, or low/no-calorie beverages may be an effective strategy for reducing energy intake [33]. There is evidence that children who consumed fruit as a snack to the point of feeling full, consumed less kJ compared to a snack of sweet biscuits or chips [34]. In Mexico, it has been shown that an increase in the price of SSBs is associated with an increase in consumption of water [35]. Reduction in the portion size of discretionary food consumed could also be an effective option in reducing intake in both children and adults [36,37,38].

Policy options that could be used to achieve the modelled benefits of reducing discretionary foods need to be considered. There are several opportunities to influence the diets of Australians and potentially reduce the consumption of SSBs and discretionary foods. A health levy to increase the price of sugary drinks has already been introduced in many countries, with evidence from Mexico that it has reduced consumption of SSBs by 10% two years after its introduction [35]. Mexico also has an 8% tax on “non-essential foods” including biscuits and cereal bars, and evaluations suggest that purchases have been reduced by between 5% and 12% [39]. A modelling study from the UK predicts that a tax and the subsequent increase in the price of high sugar snacks (biscuits, cakes, and confectionery) would result in double the reductions in BMI when compared to an increase in the price of SSBs [40]. The potential effectiveness of taxes on SSBs and discretionary foods has been demonstrated, however, health promotion initiatives may be easier to implement than strategies requiring legislation. Given the high consumption of SSBs across most age groups, reduced consumption could be a target for mass media campaigns. The LiveLighter^®^ mass media campaign has had some success in reducing the consumption of SSBs in high volume consumers [41].

Our assumptions around serving sizes of discretionary food were based on weights and volumes from the ADGs and may not reflect the serves actually being consumed by Australians. This appears to be the case particularly for cakes and chocolates, where the ADGs have assumed the serving sizes are roughly 500–600 kJ. Therefore a serve of cake is assumed to be 40 g [4], however this translates to consumers of cake in all age groups having 2–3 serving sizes per day. This indicates that the ADGs are more a recommendation for a serving size and may underestimate the actual amount consumed as a serve for specific discretionary foods. Similarly, a serving size of chocolate in the ADGs is 30 g and teenagers are consuming 15 serves per week of chocolate which equates to at least an average sized chocolate bar, such as a Mars bar (53 g) or Kit Kat (45 g), per day. If we assumed larger serving sizes in our modelling, then a single serve reduction of discretionary foods would produce greater benefits than our current modelling predicts.

It should be noted that we have only included the reduced kJ benefits of food and drink substitutes. We have not included the other health benefits, for example, the dietary fibre and vitamins in fruits, nor the reduction in negative health impacts of high sugar, sodium, and saturated fats from discretionary foods [4].

Decision makers require analyses based on the costs and benefits of potential policy options [42]. However, our study is based on hypothetical scenario modelling and is not a full cost-effectiveness analysis as it does not consider the cost of implementing interventions to achieve the reduction in consumption of the modelled discretionary foods. We acknowledge the limitations of simulation modelling but believe these results to be the best estimate in the absence of direct evidence. Additionally, we only assessed the potential obesity-related impacts of reducing discretionary food, but recognise that dental health benefits from reduced sugar intake are also likely. Some limitations of using the AHS as the basis of the consumption of discretionary foods in Australia are also acknowledged. Respondents take the survey over two days and it therefore may not capture typical daily food consumption, however we consider this to be the best available evidence.

In conclusion, we estimate that small reductions in discretionary food, particularly SSB and sweet biscuit consumption, can have significant population level health impacts and substantial healthcare cost savings. The results provide useful information for public health practitioners and policy makers to design specific health promotion programs and policies that aim to target the key foods that contribute most to obesity-related disease in the Australian population.

## Figures and Tables

**Table 1:** Serving sizes of foods modelled and uncertainty ranges.

Food and Drink Categories	Serving Size	Mean kJ Per Serve (Standard Error) *	Uncertainty Distribution	Number of Items from AHS
Sugar-sweetened beverages e.g., soft drinks, flavoured mineral water, sports drinks, cordial #	375 ml (e.g., 1 standard can)	633 (10)	lognormal	45
Cakes e.g., muffins, scones, cake-type desserts, doughnuts #	40 g (e.g., 1 slice)	597 (7)	lognormal	186
Sweet biscuits #	35 g (e.g., 2–3 biscuits)	676 (7)	lognormal	79
Frozen milk products e.g., ice-cream, frozen yoghurt, gelato #	75 g (e.g., 2 scoops)	639 (20)	lognormal	62
Chocolate e.g., chocolate bars #	30 g (e.g., ½ bar)	509 (8)	lognormal	57
Muesli bars e.g., cereal, nut, fruit or seed bars #	40 g (e.g., 1 bar)	702 (15)	lognormal	40
Salty snacks e.g., crisps, salty crackers #	30 g (e.g., ½ snack pack size)	622 (8)	lognormal	41
Confectionery e.g., lollies #	40 g (e.g., 5–6 small lollies)	606 (15)	lognormal	26
Fruit e.g., fresh or canned #	150 g (e.g., one medium, two small pieces or one cup chopped)	330 (48)	lognormal	7
Popcorn *	15 g (e.g., 2 cups)	230	-	-
Coffee e.g., flat white or latte #	250 ml	295 (23)	lognormal	64
Tea e.g., with milk, chai latte, herbal #	250 ml	218 (80)	lognormal	19
Milk plain e.g., cows, soy (reduced fat) #	250 ml	495 (8)	lognormal	-

Table notes: g = grams, ml = millilitres, Sources: AHS; Australian Health Survey 2011, AUSNUT 2011–2013 data files [21], * Live Lighter^®^ website [22], # NUTTAB Australia Food Composition Database [23].

**Table 2:** Proportion of consumers of discretionary foods, daily consumption (kJ), and energy intake reductions modelled (kJ).

Age (Years)		SSBs	Sweet Biscuits	Cakes	Chocolate	Salty Snacks	Confectionery	Muesli Bars	Frozen Milk Products
2 to 4	Proportion of consumers	40%	44%	21%	19%	22%	21%	18%	24%
	Mean kJ per day (consumers)	323	548	1380	549	612	198	552	549
	Mean kJ per day (population)	128	240	294	107	137	42	102	132
	kJ reduction per day (population) - scenario 1 *	8	22	8	5	13	8	10	11
	kJ reduction per day (population) - scenario 2 *	36	42	18	14	20	18	18	22
5 to 12	Proportion of consumers	55%	41%	28%	26%	39%	20%	22%	31%
	Mean kJ per day (consumers)	409	684	1856	747	763	333	628	933
	Mean kJ per day (population)	226	282	523	196	298	68	140	289
	kJ reduction per day (population) - scenario 1 *	11	20	11	7	22	8	12	14
	kJ reduction per day (population) - scenario 2 *	50	40	24	19	35	18	22	28
13 to 18	Proportion of consumers	64%	27%	22%	26%	28%	16%	16%	24%
	Mean kJ per day (consumers)	573	864	1938	1086	956	411	659	1103
	Mean kJ per day (population)	364	232	426	285	271	65	108	270
	kJ reduction per day (population) - scenario 1 *	13	13	8	7	16	6	9	11
	kJ reduction per day (population) - scenario 2 *	57	26	19	19	25	14	17	22
19 to 24	Proportion of consumers	58%	16%	18%	21%	19%	11%	11%	16%
	Mean kJ per day (consumers)	615	1101	1965	869	1041	653	810	1161
	Mean kJ per day (population)	354	181	361	182	196	73	93	191
	kJ reduction per day (population) - scenario 1 *	31	8	7	5	11	4	6	7
	kJ reduction per day (population) - scenario 2 *	52	16	16	15	17	10	12	15
25 to 34	Proportion of consumers	49%	23%	18%	24%	17%	10%	10%	17%
	Mean kJ per day (consumers)	603	729	2056	913	1050	437	747	1017
	Mean kJ per day (population)	296	167	378	215	177	43	75	170
	kJ reduction per day (population) - scenario 1 *	26	11	7	6	9	4	5	7
	kJ reduction per day (population) - scenario 2 *	44	22	16	17	15	8	10	15
35 to 44	Proportion of consumers	38%	23%	22%	23%	15%	10%	11%	15%
	Mean kJ per day (consumers)	573	648	1972	905	890	434	722	877
	Mean kJ per day (population)	218	152	438	211	134	45	76	130
	kJ reduction per day (population) - scenario 1 *	20	12	8	6	8	4	6	7
	kJ reduction per day (population) - scenario 2 *	34	23	19	17	13	9	11	14
45 to 54	Proportion of consumers	30%	23%	23%	22%	12%	11%	8%	15%
	Mean kJ per day (consumers)	562	665	2028	943	970	446	744	1021
	Mean kJ per day (population)	171	155	457	210	120	49	56	156
	kJ reduction per day (population) - scenario 1 *	16	12	9	6	7	4	4	7
	kJ reduction per day (population) - scenario 2 *	28	23	19	16	11	10	8	14
55 to 64	Proportion of consumers	29%	28%	24%	21%	8%	11%	6%	18%
	Mean kJ per day (consumers)	479	652	1608	949	715	361	703	917
	Mean kJ per day (population)	137	185	387	199	58	40	40	164
	kJ reduction per day (population) - scenario 1 *	15	14	9	5	5	4	3	8
	kJ reduction per day (population) - scenario 2 *	26	27	21	15	7	10	6	16
65 to 74	Proportion of consumers	25%	37%	29%	20%	7%	11%	3%	21%
	Mean kJ per day (consumers)	394	550	1689	557	617	390	920	788
	Mean kJ per day (population)	100	203	482	111	46	43	24	166
	kJ reduction per day (population) - scenario 1 *	14	18	11	5	4	4	1	9
	kJ reduction per day (population) - scenario 2 *	23	36	24	14	7	10	3	19
75 and over	Consumers	27%	42%	28%	17%	5%	12%	2%	25%
	Mean kJ per day (consumers)	375	542	1681	580	820	340	763	790
	Mean kJ per day (population)	102	230	472	100	41	41	15	200
	kJ reduction per day (population) - scenario 1 *	15	21	11	4	3	5	1	11
	kJ reduction per day (population) - scenario 2 *	25	41	24	13	4	10	2	23
population	Consumers	40%	29%	23%	22%	5%	13%	10%	20%
	Mean kJ per day (consumers)	527	681	1850	860	861	402	678	908
	Mean kJ per day (population)	211	200	432	192	143	52	69	180
	kJ reduction per day (population) - scenario 1 *	21	15	9	6	9	5	5	9
	kJ reduction per day (population) - scenario 2 *	36	28	20	16	15	11	10	18

Notes: kJ kilojoule; * scenario 1 reduction of 1 serving size per week with substitution, scenario 2 reduction of 1 serving size per week without substitution; SSBs: sugar sweetened beverages. Variability around each of these inputs (95% CI) were incorporated into the modelled results, however have not been shown.

**Table 3:** Weight reduction (kg) from reduction in each category of discretionary food by age (years) (scenario 1).

Age (Years)	kg Reduction from SSBs (UI)	kg Reduction from Sweet Biscuits (UI)	kg Reduction from Cakes (UI)	kg Reduction from Chocolate (UI)	kg Reduction from Salty Snacks (UI)	kg Reduction from Confectionery (UI)	kg Reduction from Muesli Bars (UI)	kg Reduction from Frozen Milk Products (UI)
2 to 4	0.03 (0.02–0.04)	0.09 (0.06–0.11)	0.03 (0.02–0.05)	0.02 (0.01–0.03)	0.05 (0.04–0.06)	0.03 (0.02–0.05)	0.04 (0.03–0.05)	0.04 (0.03–0.06)
5 to 12	0.06 (0.05–0.08)	0.11 (0.08–0.14)	0.06 (0.04–0.08)	0.04 (0.02–0.06)	0.12 (0.11–0.13)	0.04 (0.03–0.06)	0.07 (0.05–0.09)	0.08 (0.05–0.10)
13 to 18	0.11 (0.08–0.13)	0.11 (0.08–0.15)	0.07 (0.04–0.10)	0.06 (0.02–0.09)	0.14 (0.12–0.15)	0.05 (0.03–0.07)	0.08 (0.05–0.10)	0.09 (0.06–0.13)
19 to 24	0.31 (0.23–0.37)	0.08 (0.05–0.11)	0.07 (0.04–0.10)	0.05 (0.02–0.08)	0.11 (0.09–0.12)	0.04 (0.02–0.06)	0.06 (0.04–0.08)	0.07 (0.04–0.10)
25 to 34	0.26 (0.20–0.31)	0.11 (0.08–0.14)	0.07 (0.04–0.09)	0.06 (0.02–0.09)	0.09 (0.08–0.11)	0.04 (0.02–0.05)	0.05 (0.04–0.07)	0.07 (0.05–0.10)
35 to 44	0.20 (0.15–0.24)	0.12 (0.08–0.15)	0.08 (0.05–0.11)	0.06 (0.02–0.09)	0.08 (0.07–0.09)	0.04 (0.02–0.06)	0.06 (0.04–0.07)	0.07 (0.04–0.09)
45 to 54	0.16 (0.12–0.20)	0.11 (0.08–0.15)	0.09 (0.05–0.11)	0.06 (0.02–0.08)	0.07 (0.06–0.08)	0.04 (0.03–0.06)	0.04 (0.03–0.05)	0.07 (0.04–0.09)
55 to 64	0.15 (0.11–0.18)	0.14 (0.10–0.18)	0.09 (0.05–0.12)	0.05 (0.02–0.08)	0.05 (0.03–0.05)	0.04 (0.03–0.06)	0.03 (0.02–0.04)	0.08 (0.05–0.11)
65 to 74	0.14 (0.10–0.17)	0.18 (0.12–0.23)	0.11 (0.07–0.15)	0.05 (0.02–0.08)	0.04 (0.03–0.05)	0.04 (0.02–0.06)	0.01 (0.01–0.02)	0.09 (0.06–0.12)
75 to 100	0.14 (0.11–0.18)	0.21 (0.14–0.27)	0.11 (0.06–0.14)	0.04 (0.02–0.07)	0.03 (0.02–0.04)	0.05 (0.03–0.07)	0.01 (0.01–0.02)	0.11 (0.07–0.15)
Overall	0.21 (0.16–0.25)	0.12 (0.08–0.17)	0.09 (0.01–0.18)	0.05 (0.02–0.09)	0.09 (0.08–0.10)	0.04 (0.02–0.06)	0.04 (0.03–0.08)	0.07 (0.06–0.09)

Notes: kg: kilograms, SSBs: sugar sweetened beverages, UI: Uncertainty interval.

**Table 4:** Incident cases prevented, health adjusted life years (HALYs) gained and cost savings (scenario 1) from reducing each category of discretionary food.

Cases Prevented	SSBs (95% UI)	Sweet Biscuits (95% UI)	Chocolate (95% UI)	Confectionery (95% UI)	Salty Snacks (95% UI)	Muesli Bars (95% UI)	Cakes (95% UI)	Frozen Milk Products (95% UI)
Diabetes Type 2	31,629 (22,757–40,495)	26,107 (15,479–36,968)	10,023 (3649–16,926)	7949 (4396–12,085)	10,559 (8567–12,587)	6105 (3382–9327)	17,708 (1397–36,305)	14,224 (11,729–16,848)
Osteoarthritis knee and hip	17,125 (10,945–23,668)	12,662 (7218–19,311)	6100 (2186–10,808)	4675 (2306–7724)	7019 (4837–9400)	4315 (2176–7153)	9596 (713–20,534)	6756 (4773–88,190)
Ischemic heart disease	10,206 (7536–12,426)	11,810 (7492–16,227)	3211 (1195–5276)	2957 (1687–4382)	2491 (2149–2859)	1151 (661–1718)	7130 (571–14,305)	6251 (5789–6699)
Stroke	3455 (2267–4678)	4876 (2894–7111)	1070 (394–1894)	1110 (575–1767)	620 (383–881)	188 (78–329)	2714 (254–5658)	2480 (1884–3052)
Breast cancer	784 (325–1281)	1044 (446–1832)	301 (90–599)	293 (106–543)	239 (98–381)	115 (43–215)	613 (44–1498)	446 (207–701)
Colorectal cancer	784 (397–1144)	913 (400–1541)	204 (62–410)	188 (67–342)	105 (16–201)	27 (9–69)	571 (48–1325)	581 (371–791)
Endometrial cancer	579 (241–1890)	750 (−324–2657)	215 (−107–854)	215 (−794–106)	178 (75–566)	90 (−39–323)	436 (−236–2062)	334 (−152–1047)
Hypertensive heart disease	606 (418–800)	809 (509–1154)	172 (64–298)	177 (98–270)	89 (55–129)	25 (11–44)	470 (39–971)	464 (369–562)
Kidney cancer	501 (315–713)	634 (362–953)	165 (59–298)	156 (81–246)	116 (78–158)	48 (23–79)	378 (33–787)	329 (222–435)
Total HALYs	76,441 (57,214–94,597)	66,550 (41,702–91,563)	24,787 (9098–40,778)	19,959 (11,228–29,826)	25,397 (21,934–29,545)	14,339 (8084–21,917)	44,711 (3907–90,169)	35,616 (32,082–39,341)
Healthcare cost savings ($M)	793.4 (589.1–976.0)	640.7 (402.6–885.8)	260.0 (95.8–432.8)	203.8 (113.3–305.7)	280.8 (243.0–320.6)	163.9 (92.9–249.4)	447.1 (38.3–903.2)	345.4 (310.3–381.9)

Notes: HALYs, health adjusted life years; M, Millions; UI, uncertainty interval.

## Supplementary Materials

The following are available online at https://www.mdpi.com/2072-6643/12/3/649/s1, Table S1: Australian Health Survey food classification codes; Table S2: Results of scenario 2: One serve reduction per week (no substitution): incident cases prevented, HALYs and cost savings.
