# Supplementary material for: The Modelled Population Obesity-Related Health Benefits of Reducing Consumption of Discretionary Foods in Australia
Source: Nutrients. 2020 Feb 28;12(3):649. doi: 10.3390/nu12030649 (PMC7146305; doi:10.3390/nu12030649)
Supplement: Supplementary file 1 [file nutrients-12-00649-s001.pdf]

Table 1. Australian Health Survey food classification codes [20].

Discretionary food/ drink	Food group codes
SSBs	114,115,116,
Cakes	133
Sweet biscuits	131
Ice cream	195
Chocolate	281
Muesli bars	283
Salty snacks	26
Confectionery	284

Table 2. Results of scenario 2: One serve reduction per week (no substitution): incident cases prevented, HALYs and cost savings.

	SSBs (95%UI)	Sweet biscuits (95%UI)	Chocolate (95%UI)	Confectionery (95%UI)	Salty snacks (95%UI)	Muesli bars (95%UI)	Cakes (95%UI)	Frozen milk products (95%UI)
**Cases prevented**								
Diabetes Type 2	53,768 (36,767–73,561)	52,333 (40,802–65,042)	28,353 (21,785–35,942)	17,561 (13,334–22,294)	16,441 (10,836–20,707)	11,562 (8562–14,988)	39,996 (23,744–60,268)	32,458 (21,631–462,320)
Osteoarthritis knee and hip	29,258 (18,384–42,890)	25,512 (16,835–34,947)	17,419 (11,487–24,504)	10378 (6,545–14,772)	10,973 (4301–14,915)	8252 (5265–11,853)	21,814 (11,570–35,177)	15,713 (9,234–23,986)
Ischemic heart disease	17,439 (12,519–23,087)	23,768 (19,715–27,808)	9163 (7461–11,159)	6553 (5376–7862)	3883 (2754–4622)	2,198 (700–2734)	16,096 (9,772 –23,288)	14,167 (9,794—19,228)
Stroke	5,860 (3774–8341)	9829 (7100–12,815)	3036 (2085–4108)	2450 (1677–3309)	969 (372–1359)	359 (561–177)	6191 (3484–9615)	5604 (3616–8103)
Breast cancer	1335 (565–2283)	2,102 (944–3356)	856 (387–1381)	646 (283–1061)	376 (20–608)	222 (96–366)	1375 (548 –2,59)	1054 (460–1823)
Colorectal cancer	1286 (651–2087)	1817 (880–2849)	585 (250–960)	417 (166–681)	163 (-51–307)	52 (-15–129)	1293 (600–2230)	1282 (689–2044)
Endometrial cancer	929 (−475–3358)	1568 (−757–4921)	616 (−296—2,022)	492 (−188–1541)	278 (−121–879)	170 (−71–560)	1014 (−447–3703)	766 (−357–2657)
Hypertensive heart disease	1034 (685–1456)	1629 (1251–2030)	490 (355–633)	390 (294–506)	139 (66–187)	48 (25–73)	1058 609–1596	1041 (674–1485)
Kidney cancer	862 (510–1286)	1277 (877–1698)	464 (310–632)	343 (232–467)	178 (69–247)	92 (57–133)	854 (461–1356)	749 (451–1151)
**HALYs**	129,997 (92,532–173,013)	134,168 (110,377–159,044)	70,320 (56,046–86,487)	44,171 (35,601–54,169)	39,617 (31,768–48,726)	27,263 (20,814–34,711)	101,280 (60,645–149,939)	81,114 (55,542–111,081)
**Healthcare savings $M**	1,350.5 (959.3–1769.4)	1,288.7 (1.051.6–1.530.9)	739.4 (591.2–919.1)	450.8 (357.8–550.8)	438.4 (314.4–532.6)	311.9 (238.0–399.6)	1,013.0 (610.6–1490.0)	790.7

	(536.6–1,1089.<br>1)
